# Age-Related Changes in Immune Cells of the Human Cochlea

**DOI:** 10.3389/fneur.2019.00895

**Published:** 2019-08-16

**Authors:** Kenyaria V. Noble, Ting Liu, Lois J. Matthews, Bradley A. Schulte, Hainan Lang

**Affiliations:** ^1^Department of Pathology and Laboratory Medicine, Medical University of South Carolina, Charleston, SC, United States; ^2^Department of Otolaryngology-Head and Neck Surgery, Medical University of South Carolina, Charleston, SC, United States

**Keywords:** hearing loss, macrophage, aging, human temporal bone, cochlea

## Abstract

Age-related hearing loss is a chronic degenerative disorder affecting one in two individuals above the age of 75. Current population projections predict a steady climb in the number of older individuals making the search for interventions to prevent or reverse this disorder even more critical. There is growing acceptance that aberrant activity of resident or infiltrating immune cells, such as macrophages, is a major factor contributing to the onset and progression of age-related degenerative diseases. However, how macrophage populations and their functionally-driven morphological characteristics change with age in the human cochlea remains largely unknown. In this study, we employed immunohistochemical approaches along with confocal and super-resolution imaging, three-dimensional reconstructions, and quantitative analysis to determine age-related changes in macrophage numbers and morphology as well as interactions with other cell-types and structures of the auditory nerve and lateral wall in the human cochlea. In the cochlea of human ears from young and middle aged adults those macrophages in the auditory nerve assumed a worm-like structure in contrast to those in the spiral ligament or associated with the dense microvascular network in the stria vascularis which exhibited a highly ramified morphology. Macrophages in both the auditory nerve and cochlear lateral wall showed morphological alterations with age. The population of activated macrophages in the auditory nerve increased in cochleas obtained from older donors. Dual-immunohistochemical staining with macrophage, myelin, and neuronal markers revealed increased interactions of macrophages with the glial and neuronal components of the aged auditory nerve. These findings implicate the involvement of abnormal macrophage-glia interactions in age-related physiological and pathological alterations in the human cochlea. There is clearly a need to further investigate the contribution of macrophage-associated inflammatory dysregulation in human presbyacusis.

## Introduction

Age-related hearing loss (ARHL), or presbyacusis, is one of the most prevalent chronic disorders affecting the older adult population in the US, with one in three individuals over the age of 60 and one in two individuals over the age of 75 reporting moderate-to-severe hearing loss (1). ARHL is characterized by reduced hearing sensitivity as measured by pure-tone thresholds with individuals often complaining of difficulties understanding speech, especially in noisy environments (2). This reduction in hearing may lead to quality of life changes such as social isolation and depression (3).

The most common treatment recommendation for ARHL is hearing aids. For those with moderate-to-profound hearing loss who no longer receive benefit from hearing aids, cochlear implants may be recommended. Cochlear implants are surgically placed devices which electrically stimulate the nerves of the inner ear directly. It has been shown that cochlear implantation may induce activation of both immune and non-immune cells, and thereby have a negative impact on the outcome of this therapeutic approach (4). Interrogation of the cochlear inflammatory response to implantation in a mouse model identified a significant up-regulation in the expression of pro-inflammatory cytokines such as IL-1β. A better understanding of the specific cellular and molecular mechanisms associated with ARHL, in particular the role of the immune system, is necessary to identify targetable cells and pathways for the development of new and novel approaches of remediation.

There is now growing acceptance that aberrant immune cell activity is a contributive factor in the onset and progression of age-related degenerative diseases (5, 6). This includes disorders such as cardiovascular disease, which has high prevalence among older individuals and is co-morbid with ARHL (7). Characterization of immune cell activity in cardiovascular disease pathology indicates atherosclerotic plaque formation is associated with the phagocytic uptake of lipid particles by macrophages (6, 8). Dementia, another chronic disorder which shows strong correlation with ARHL (9), is characterized by neurodegeneration in which the dysregulation of immune cell activity is a promising therapeutic target (5). For example, in Alzheimer's disease, reduced degradation activity by microglia has been shown to contribute to the accumulation of amyloid-beta plaques and neurofibrillary tangles (10), which can be ameliorated by the presence of young microglial cells (11).

Previous studies on the cellular and molecular processes which contribute to ARHL have focused primarily on degenerative changes in sensory hair cells (12–14) and the auditory nerve (15, 16). Immunohistochemical analysis of immune cell distribution in the mouse and human cochlea, indicates that macrophages are largely excluded from the healthy adult organ of Corti, the cochlear region housing the inner and outer hair cells (17). Other studies have provided evidence demonstrating not only the presence of resident macrophages in the cochlear lateral wall and auditory nerve but also their direct interaction with the strial microvessels and spiral ganglion neurons in the human ear (18–20). Furthermore, recent studies indicate that activated microglia play an important role in the disruption of the blood-brain barrier and demyelination in the central nervous system (21, 22).

Based on the above observations, we hypothesized that macrophage function and activity in the cochlea changes with age and these changes may be a contributive factor to pathophysiological alterations of cochlear structures in ARHL. Here we have addressed this hypothesis by evaluating changes in the number and morphology of macrophages, together with observations of interactions between activated macrophages and other cochlear cell types and structures in the lateral wall and auditory nerve in an age-graded series of human inner ears.

## Materials and Methods

### Collection and Preparation of Cochlear Tissues

Human temporal bone samples were obtained from two sources; (1) the Medical University of South Carolina (MUSC) Hearing Research Program's temporal bone collection generated from a longitudinal study of ARHL and (2) the MUSC Carroll A. Campbell, Jr. Neuropathology Laboratory Brain Bank. In all cases of HTB collection, written and informed consent was obtained from the next-of-kin in accordance with South Carolina laws and regulations. Temporal bone research was approved by the MUSC Institutional Review Board as not human subject research (Pro0030845). Table 1 lists the donors' age, sex, and the time between death and fixation for the 12 human temporal bones used in this study. After removal of the specimen (23), scalar perfusion was performed with a 4% solution of paraformaldehyde as previously described (24, 25) and fixation was continued by immersion for 48–72 h. The bones were then rinsed with phosphate-buffered saline (PBS) and decalcified in EDTA, for a period of 4–6 weeks as previously described (26, 27). Over this period, the specimens were trimmed to remove the hard bone covering the cochlea and vestibular apparatus of the inner ear. The inner ear portion of the trimmed temporal bones was processed for frozen sectioning and whole mount preparations as described previously (25, 28, 29).

**Table 1:** Human temporal bone donor metrics.

**Group**	**ID**	**Age**	**Sex**	**Postmortem fixation interval (hours)**
Younger (*n* = 5)	H41	20	M	23.5
	H98	31	M	7.0
	H109	42	M	8.7
	H87	55	F	5.8
	H107	65	M	5.3
Older (*n* = 7)	H38	68	F	6.5
	H94	69	F	5.4
	H114	75	F	3.5
	H55	86	M	4.8
	H33	87	F	3.6
	H51	89	F	35.0
	H34	>89	F	3.3

For all analyses, the human cochlea samples were divided into two groups: younger group ranging in age from 20 to 65 years and older group ranging in age from 68 to >89 years (older).

### Immunostaining and Quantitative Analysis of IBA1^+^ Cells

The whole mounts and frozen sections of inner ear tissue were subjected to immunofluorescence staining as described briefly below. Whole mounts were washed with PBS and incubated in 4:1:1 (methanol: 30% hydrogen peroxide: dimethyl sulfoxide) solution for 1 h at room temperature prior to staining. The tissues/samples were incubated overnight at 4°C with a primary antibody (Table 2) diluted in 0.2% bovine serum albumin (BSA) in PBS. After rinsing with PBS, the appropriate biotinylated secondary antibody was applied to the sections followed by conjugation with Fluorescein-labeled (A-2011, Vector), Texas Red-labeled (A-2006, Vector) avidin, or a DAB immunoperoxidase secondary detection system (DAB150, Millipore). For dual-labeling, sections were processed using an avidin/biotin blocking kit (SP-2001, Vector) following the first staining reaction, according to the manufacturer's instructions. After a 30-min incubation in 0.2% BSA in PBS, staining was continued overnight by incubation with a second primary antibody. Nuclear counterstaining was performed with either propidium iodide (PI) or 4′,5-diamidino-2-phenylindole (DAPI).

**Table 2:** Antibodies and other reagents used for immunohistochemistry.

**Material**	**Company**	**Catalog no**.	**Dilution**
Rabbit anti-IBA1	Wako	019-19741	1:200
Chicken anti-MBP	EMD Millipore	AB9348	1:100
Mouse anti-NF	Sigma	N0142	1:200
Mouse anti-CD163	Invitrogen	MA5-17716	1:200
Rabbit anti-Kir4.1	Alomone Labs	PC035AN0802	1:100
Biotinylated goat anti-Rabbit IgG	Vector	BA-1000	1:150
Biotinylated goat anti-Chicken IgY	Vector	BA-9010	1:150
Fluorescein Avidin D	Vector	A-2011	1:150
Texas Red Avidin D	Vector	A-2006	1:150
Propidium Iodide	Millipore Sigma	P4864	1:1,000
Hoechst 33342	Millipore Sigma	B2261	1:1,000

The numbers of IBA1^+^ cells located in the cochlear lateral wall and the auditory nerve within Rosenthal's Canal were quantified on randomized sections (2–4 sections per sample) by researchers blinded to specimen age. In this study, cell counting was performed on the frozen cochlear sections that were developed with a fluorescence staining approach, but not the DAB immunoperoxidase method. The use of the immunofluorescence staining, together with PI or DAPI nuclear counterstaining and confocal microscope, is the best approach to (1) identify the nuclear location of IBA1^+^ macrophages, which is the crucial step of the macrophage count, and (2) to determine how macrophages interact with other cochlear cell types if needed. For the lateral wall, both the number of IBA1^+^ cells and the number of processes per cell were quantified in the apical, middle, and basal turns of the cochlea at 40X magnification. For the auditory nerve, numbers of IBA1^+^ cells were quantified only in the middle turn. Statistical analysis of the collected data was performed with SPSS Statistics (v25, IBM) using Shapiro-Wilk normality test to confirm the data was normally distributed. Younger vs. older adult group means were compared by Student's unpaired one-tailed *t-*test.

Confocal image stacks of frozen sections and whole mount tissues were acquired using a Zeiss LSM 880 NLO microscope with Zen acquisition software (Zeiss). Image stacks were composed of 1,024 pixels (x) by 1,024 pixels (y) taken at 0.5–1 μm intervals throughout the optical plane. For super-resolution imaging, confocal image stacks (0.2 μm intervals) of frozen sections were acquired using a Zeiss LSM 880 NLO microscope with Airyscan super-resolution detector and a 63x/1.4 Plan-Apochromat oil objective (Zeiss). Multi-color images were acquired using the following laser and filter combinations: Channel 1–561 nm laser excitation and BP 570–620 + LP 645; Channel 2–488 nm laser excitation and BP 420–480 + BP 495–550.

Image processing was performed using Zen Black or Zen 2012 Blue Edition (Carl Zeiss Microscopy), Adobe Photoshop CC (Adobe Systems), and GIMPshop (open source software).

## Results

### Morphological Features of Macrophages Vary Between the Cochlear Lateral Wall and Auditory Nerve

**Figure 1:**
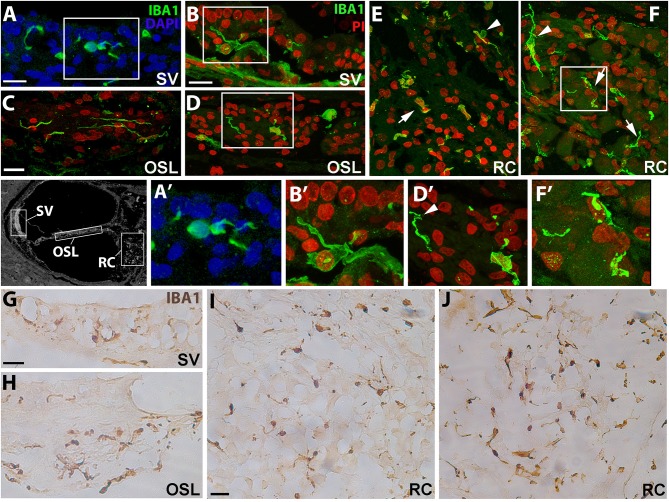
Cochlear macrophages demonstrate unique morphologies in different regions of the human cochlea. Confocal imaging of immunofluorescence staining for ionized calcium-binding adaptor molecule-1 (IBA1^+^, green) identifies resident macrophages in the stria vascularis (**A,B**—SV), osseous spiral lamina (**C,D**—OSL), and Rosenthal's canal (**E,F**—RC). Similar results were also revealed by peroxidase-DAB immunohistochemistry assay showing IBA1^+^ macrophages in the SV **(G)**, OSL **(H)**, and RC **(I,J)**. Images in **(A′,B′,D′,F′)** are enlargements of the boxed areas in **(A,B,D,F)**, respectively. The black and white image in the left of **(A′)** was included to show different cochlear locations. The image was taken from a cochlear section that was stained for Kir4.1 antibody (30) and the section was obtained from an 86-year-old donor (H55). Strial macrophages **(A,B,G)** emit cellular processes that extend short distances from the cell body **(A′,B′)**. Macrophages in the OSL **(C,D,H)** are more worm-like in shape with thin cellular projections that sometimes wrap around structures (**D′**, arrowhead), most likely peripherally projecting axons, whereas macrophages in RC **(E,F,I,J)** typically have an elongated appearance (arrows); IBA1^+^ macrophages with multiple processes are also seen (arrowheads). Images were obtained from a 42-year-old donor (H109; **A,C,E**), a 55-year-old donor (H87; **B,D,F,I**) and a 57-year-old donor (H122; **G,H,J**). Locations in the cochlea are indicated on the cochlear map by a black and white image at the middle left. Scale bars in **(A,B)** = 20 μm; in **(C)** applies to **(D–F)** = 20 μm; in **(G)** applies to **(H)** = 30 μm, in **(I)** applies to **(J)** = 30 μm.

To assess the morphology of resident macrophages in the human cochlea, we performed immunofluorescence staining using an antibody against the microglia/macrophage specific protein, ionized calcium binding adaptor molecule 1 (IBA1). In young and middle-aged ears, marked differences in morphological characteristics were seen between macrophages in the stria vascularis (SV; Figures 1A,B) and those associated with the auditory nerve both in the osseous spiral lamina (Figures 1C,D) and Rosenthal's canal (Figures 1E,F). Macrophages in the SV from 42- and a 55-year-old donor were characterized by cellular processes extending into the areas between marginal and basal cells, near the strial microvasculature (Figures 1A′,B′). This observation is in good agreement with a previous study showing strial macrophages interacting with the microvasculature (18). Observations of the auditory nerve in the same cochleas revealed macrophages in the osseous spiral lamina with a more bipolar architecture (Figure 1D, at this plane of view) and flat encroaching filopodia-like structures (Figure 1D′), suggesting interactions with peripheral neural projections. In Rosenthal's canal, IBA1^+^ cells were observed with two distinct stellate forms, a less frequent form which was often oriented perpendicular to the axonal fibers (arrowhead, Figure 1F), and a more common “worm-like” cyto-architecture located near the neuronal soma (arrows, Figure 1F′). The differential characteristics of IBA1^+^ macrophages located in the auditory nerve and cochlear lateral wall were also revealed by peroxidase DAB immunohistochemistry with frozen sections (Figures 1G–J). These differences in macrophage morphology among different cochlear regions may indicate a variety of macrophage functions since each cochlear compartment contributes uniquely to auditory physiology.

**Figure 2:**
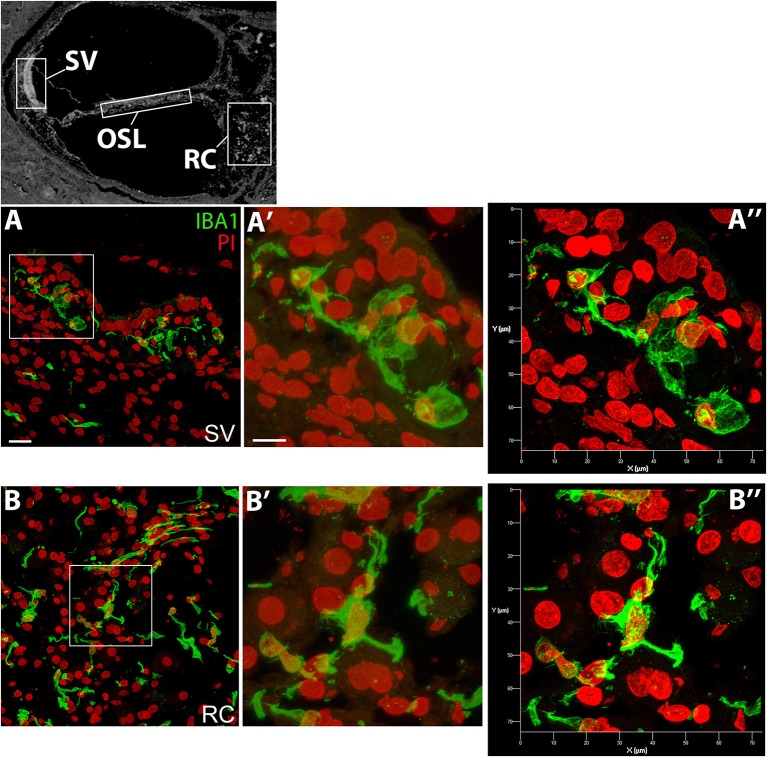
Super-resolution confocal images obtained by ZEISS Airyscan detector showing differential morphology of the IBA1+ macrophages in the SV and RC. Confocal images **(A′,B′)** and super-resolution images **(A^′′^,B^′′^)** were taken from the same boxed areas outlined in A and B. The black and white image in the top panel was included to show different cochlear locations and it is the same image used in Figure 1. All images were taken from the SV and RC of a cochlea obtained from a 57-year-old donor (H122). Scale bar in **(A)** applies to **(B)** = 20 μm; in **(A′)** applies to **(B′)** = 10 μm.

Super-resolution imaging was also applied to visualize the morphological differences of the macrophages in the auditory nerve and the lateral wall. Figure 2 includes both the standard and super resolution confocal images of IBA1^+^ macrophages taken from the same regions of the SV and auditory nerve in the middle-basal turn cochlea of a 55 year old donor. The enhancement in image quality and the power to resolve the nanoscopic filaments extending from the subcellular elements of the macrophage is clearly visualized in the super resolution images (Figures 2A^′′^,B^′′^).

**Figure 3:**
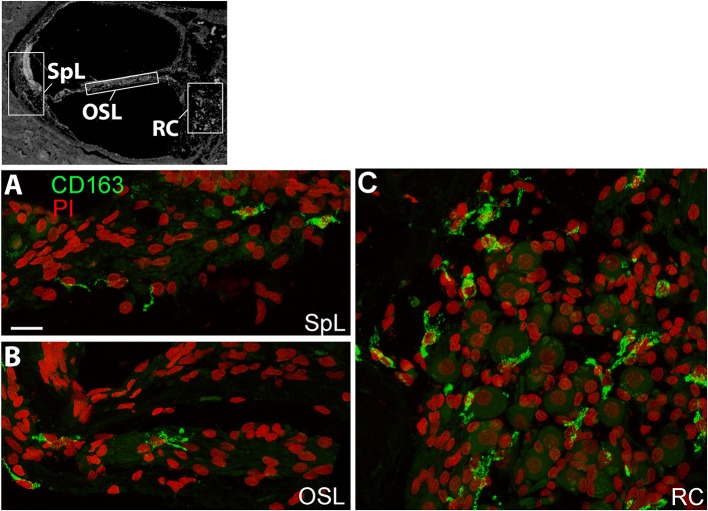
CD163^+^ macrophages are present in several regions of the human cochlea. The black and white image in the top panel was included to show different cochlear locations and it is the same image used in Figures 1, 2. Confocal imaging of immunofluorescence staining for CD163 (IBA1^+^, green) identifies resident macrophages in the spiral ligament (SpL, **A**), OSL **(B)**, and RC **(C)**. Images were obtained from a 57-year-old donor (H122). Scale bar in **(A)** applies to **(B,C)** = 20 μm.

A recent human temporal bone study characterized a number of macrophage/microglia markers, including CD163, on celloidin embedded sections of temporal bones from normal individuals (19). CD163 is a scavenger receptor expressed on the cell surface of monocytes and macrophages (31) demonstrated to be responsive to pro- and anti- inflammatory stimuli (32). In the present study, frozen sections were stained with an antibody against CD163, and we assessed immunoreactivity and distribution in different cochlear regions. CD163^+^ macrophages were observed within the spiral ligament (SpL) of the cochlear lateral wall, osseous spiral lamina, and Rosenthal's canal, as demonstrated by representative images in Figure 3. Immunostaining for CD163 appears to be less illustrative of the macrophage morphological features compared to IBA1 staining (Figure 1), thus IBA1 antibody was selected as the marker to evaluate macrophage morphology and quantitative analysis in the study.

### Diversity of Macrophages Within the Cochlear Lateral Wall

**Figure 4:**
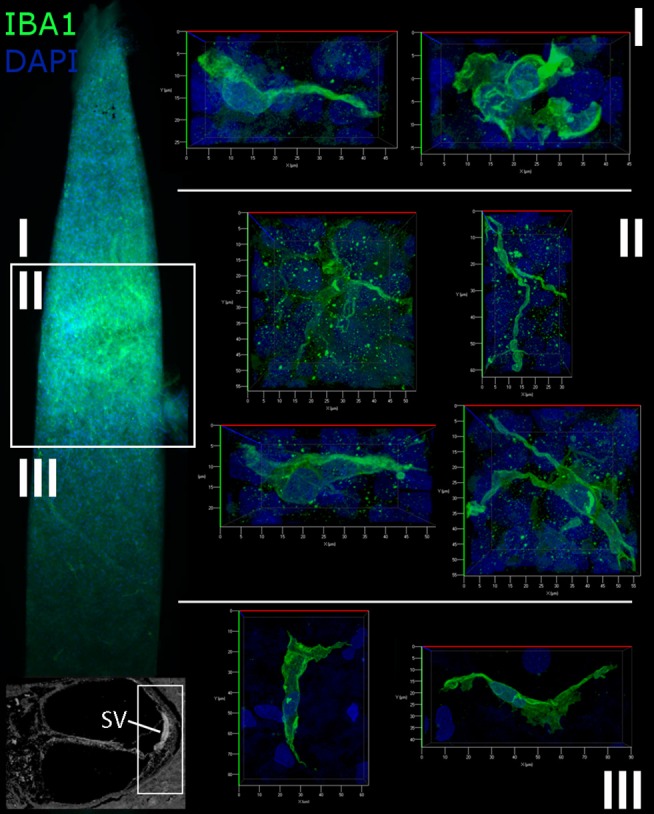
Macrophages in the human cochlear lateral wall are morphologically diverse. A whole mount preparation of the SV and SpL in the basal turn of the cochlea from a 57-year old donor (H106) was stained for IBA1 (green). The low magnification image on the left maps the suprastrial **(I)**, strial **(II)**, and substrial **(III)** regions of the lateral wall where the images were acquired. Macrophages in the SV have a stellate appearance with thin processes, while the macrophages of the suprastrial **(I)** and substrial **(III)** regions of the SpL exhibit a bipolar morphology.

Cochlear macrophage morphology was further examined in the suprastrial, strial, and substrial regions of the cochlear lateral wall by immunostaining whole mounts for IBA1 (Figure 4). This procedure allows the three-dimensional features of individual macrophages to be clearly observed. The morphological characteristics of macrophages varied among different regions of the lateral wall. In the suprastrial region of the SpL, which is typically populated by type V fibrocytes, macrophages were observed with an elongated shape and extending a single filopodia-like structure (Figure 4I), or with a rounder shape and shorter flatter cellular process (Figure 4I). In the substrial region of the SpL, which typically contains type IV fibrocytes, the IBA1^+^ cells displayed slightly extended cellular profiles in a polar morphology (Figure 4III). Macrophages in the SV exhibited a stellate appearance with numerous thin cellular extensions (“ramified” morphology; Figure 4II), a morphological profile typically associated with active surveillance of the biochemical activity in the local environment (33).

### Age-Related Changes of Macrophages in the Cochlear Lateral Wall

**Figure 5:**
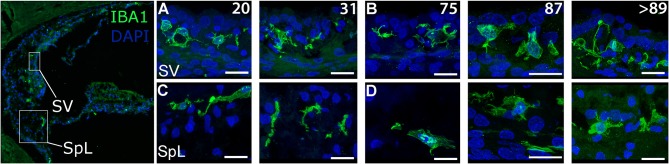
Age-related alterations in IBA1^+^ macrophage morphology and numbers in the human cochlear lateral wall. IBA1^+^ resident macrophages are present in the SV and the SpL. Images from both regions document age-related changes in macrophage morphology. IBA1^+^ cells from 20-, and a 31-year-old donar (H41 and H98, respectively) appeared to be in a “surveillance-mode” as defined by numerous processes extending from the central body **(A,C)**. In contrast, macrophages in a 75-, 87-, and >89-year-old donar (H114, H33, and H34, respectively) exhibited a rounded cellular morphology, increased cytoplasmic volume in the area surrounding the nucleus and reduced far-reaching cellular processes **(B,D)**. Scale bars in **(A–D)** = 20 μm.

Examination of the cochlear lateral wall from older donors revealed characteristic changes in IBA1^+^ macrophage morphology in the SV and SpL using both immunofluorescence staining (Figures 5A–D) and peroxidase DAB immunohistochemistry (Data not shown). In the SV of the younger donors, macrophages had little cytoplasm surrounding the nucleus and numerous projecting processes (Figure 5A). In contrast, in older donors the number of processes projecting from macrophages was reduced while cytoplasmic volume around the nucleus appeared to increase (Figure 5B). Macrophages in the adjacent SpL exhibited similar morphological alterations of increased cytoplasmic volume and reduced cellular processes in older donors (Figure 5D).

In order to quantitatively assess changes in the cellular properties of macrophages in the SpL, IBA1^+^ cells were counted in two age groups, younger vs. older donors (*n* = 5/group). The number of macrophages are reported as cell number per observed area, which included the entire cochlear turn. In the apical turn, the number of IBA1^+^ macrophages was very similar in the younger and older groups (2.7 ± 1.0 vs. 2.9 ± 1.0 cells, respectively, *p* = 0.34). Quantification of macrophage numbers in the middle (7.8 ± 3.5 vs. 10.7 ± 3.8 cells) and basal turn (11.3 ± 6.5 vs. 14.5 ± 6.1 cells) of younger vs. older ears, respectively, indicates a trending increase in both of these regions in the older group, but this change failed to reach statistical significance (*p*-values = 0.12 and 0.23, respectively). The results of cell counting from individual donors, listed in Table 3, reveal a large amount of variability in the younger control group. Activated macrophages, defined as IBA1^+^ cells with no or only a few cellular processes, were also quantified to determine if the number of activated macrophages changes with age. Table 4 lists these results from individual donors. No statistically significant differences were found. In the apical turn, the presence of activated macrophages in the SpL was unchanged (1.0 ± 1 vs. 1.0 ± 0.5 cells, *p* = 0.50); though a trending increase in the number of activated macrophages was noted for the middle (4.0 ± 3.3 vs. 6.4 ± 2.4 cells, *p* = 0.11) and basal turns (4.9 ± 3.0 vs. 7.9 ± 3.5 cells, *p* = 0.09) of the older group.

**Table 3:** Average number of macrophages in the human lateral wall and auditory nerve.

**Group**	**ID**	**Age**	**Lateral wall**			**Auditory nerve**
			**Apical**	**Middle**	**Base**	**Middle**
Younger (*n* = 5)	^*^H41	20	4.3 ± 1.5	11.3 ± 2.3	6.7 ± 2.5	20.0 ± 2.3
	H98	31	2.7 ± 0.6	11.7 ± 3.5	22.0 ± 3.6	16.0 ± 9.9
	H109	42	1.5 ± 0.7	4.5 ± 0.7	5.5 ± 0.7	12.0 ± 1.4
	H87	55	2.5 ± 1.3	4.8 ± 3.7	12.0 ± 4.1	13.0 ± 4.6
	H107	65	2.3 ± 1.2	7.0 ± 2.0	10.3 ± 2.5	9.5 ± 0.7
Older (*n* = 7)	H38	68	2.3 ± 2.3	4.7 ± 1.5	6.0 ± 1.0	17.5 ± 0.7
	H94	69	n.c.	n.c.	n.c.	20.0 ± 4.4
	H114	75	n.c.	n.c.	n.c.	13.0 ± 0.0
	^*^H55	86	4.0 ± 0.0	12.7 ± 3.1	19.3 ± 7.5	24.0 ± 4.0
	H33	87	3.7 ± 1.2	14.7 ± 2.1	19.3 ± 2.1	20.7 ± 6.4
	H51	89	1.7 ± 0.6	10.0 ± 3.6	10.0 ± 3.5	19.0 ± 1.4
	H34	>89	3.0 ± 1.0	11.7 ± 2.5	17.7 ± 2.5	19.3 ± 1.2

*The data are presented as cell number per observed area which covered an intact cochlear turn*.

* *Post-mortem fixation time >20 h*.

*n.c., not counted*.

**Table 4:** Average number of activated macrophages in the human lateral wall.

**Group**	**ID**	**Age**	**Lateral wall**		
			**Apical**	**Middle**	**Base**
Younger (*n* = 5)	H41	20	2.7 ± 1.2	9.0 ± 2.0	2.3 ± 2.3
	H98	31	1.0 ± 0.0	5.7 ± 4.0	9.3 ± 3.5
	H109	42	0.5 ± 0.7	2.0 ± 2.8	2.0 ± 1.4
	H87	55	1.0 ± 0.8	1.8 ± 3.4	6.3 ± 3.4
	H107	65	0.0 ± 0.0	1.7 ± 1.5	4.7 ± 2.1
Older (*n* = 5)	H38	68	0.7 ± 1.2	3.0 ± 1.0	4.0 ± 2.0
	H55	86	0.5 ± 0.7	6.3 ± 4.6	9.7 ± 3.5
	H33	87	1.7 ± 0.6	9.7 ± 0.6	12.7 ± 5.5
	H51	89	1.0 ± 1.0	6.0 ± 4.4	5.0 ± 1.7
	H34	>89	1.3 ± 1.1	7.0 ± 1.0	8.0 ± 2.6

*The data are presented as cell number per observed area which covered an intact cochlear turn*.

### Diverse Morphology of Macrophages in the Auditory Nerve

**Figure 6:**
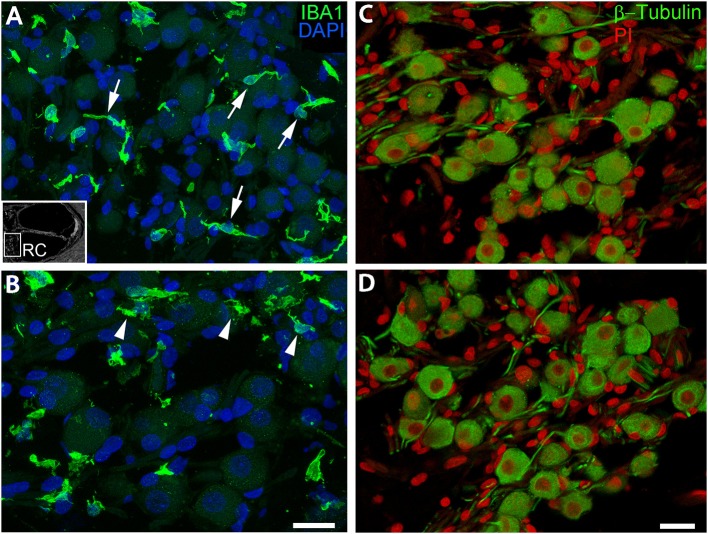
IBA1^+^ macrophages located in the auditory nerve demonstrate spatially-dependent alterations in morphology. These images from the auditory nerve (AN) of an 87-year-old donor (H33) demonstrate region-dependent changes in macrophage morphology. IBA1^+^ macrophages in the middle turn **(A)** have an elongated spiral shape (arrows) whereas many cells in the basal turn **(B)** extend numerous short cellular projections (arrowheads), although there is no evidence supporting a robust loss of β-Tubulin^+^ SGNs **(C,D)**, which appear in the same area of the AN. An inserted black and white image in the bottom-left of **(A)** shows the location of the AN in the cochlea. Scale bar in **(B)** applies to **(A)** = 20 μm; in **(D)** applies **(C)** = 20 μm.

A recent study employing super-resolution imaging in the human cochlea demonstrated that resident macrophages in the auditory nerve make contact with spiral ganglion neurons (18). Here we assessed the cellular morphology of the IBA1^+^ macrophages in the auditory nerve from an 87-year-old donor (Figure 6). In the middle turn (Figure 6A), the macrophages had thin cellular processes with little cytoplasm around the nucleus, an architecture possibly reflecting interactions with axons and neuronal cell soma. Macrophages in the basal turn from the same donor exhibit a markedly different appearance having amorphous or round shapes with less evidence of extended cellular projections (Figure 6B). Interestingly, no noticeable loss of β-Tubulin^+^ spiral ganglion neurons was identified in the basal turn of the auditory nerve of this donor (Figures 6C,D). These observations suggest the morphology and activity of macrophages may be dependent on not only cell death, but also location and physiological and/or pathological activity along the cochlear axis.

### Age-Related Changes in Number and Cellular Interactions of Macrophages in the Auditory Nerve

**Figure 7:**
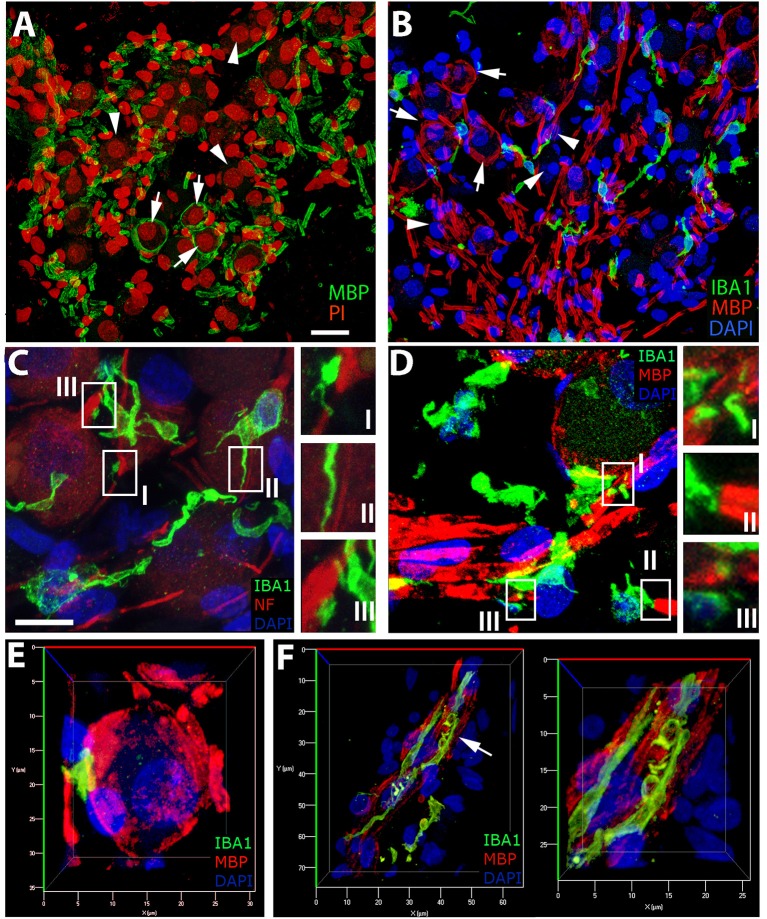
IBA1^+^cells in the auditory nerve demonstrate close contact with myelin and axons of the auditory nerve. **(A,B)** The presence of myelinated (MBP^+^, arrows) and unmyelinated (arrowheads) spiral ganglion neurons (SGNs) in the human auditory nerve is demonstrated by representative images from the basal **(A)** and middle **(B)** turns of an 87-year-old donor (H33). IBA1^+^ macrophages (green) are observed in close apposition with NF^+^ axons (**C**, red) and MBP^+^ myelin sheath (**B,D**, red) in sections from the middle turn of the same donor (H33). **(C)** Macrophages demonstrate a variety of interactions with the NF^+^ components of spiral ganglion neurons (SGNs). Macrophage processes are observed interfacing with NF^+^ axons in areas of discontinuities of the myelin sheath of SGNs (I), running parallel to the axon process (II), and in some cases directly interacting with the neural cell elements (III). **(D)** Macrophages are often observed with a “worm”-like morphology along myelinated neurons with processes terminating at areas with little or no myelin (I,III). **(E)** Three-dimensional reconstructions of confocal images from IBA1 and MBP dual-labeling provide further observations of macrophages interacting with the myelin of a Type I SGN soma in Rosenthal's canal of the cochlea obtained from the same donor (H33) shown in **(A–D)**. **(F)** Evidence of macrophage engulfment of MBP^+^ myelin in the auditory nerve in the osseous spiral lamina in the apical turn of a 42-year-old donor (H109). The right panel is the enlarged image of the region identified by an arrow in the panel at left. Scale bar in **(A)** applies to **(B)** = 20 μm; in **(C)** applies to **(D)** = 10 μm.

We have previously demonstrated the presence of myelination in the parikarya of some Type I spiral ganglion in the human auditory nerve (24) and provide further evidence of this phenomenon (Figures 7A,B,D,E) in the current study. To assess macrophage interactions with components of the human auditory nerve, we performed dual labeling immunohistochemistry. Antibodies against either neurofilament 200 (NF; Figure 7C) or myelin basic protein (MBP; Figures 7B,D–F) were used in combination with an anti-IBA1 antibody to label auditory nerve components and macrophages, respectively. Macrophages with a variety of morphologies interact with myelin or axons of the auditory nerve. The filopodia-like structures of IBA1^+^ macrophages are seen at discontinuities in the neurofilament bundles (Figure 7CI), running parallel to filamentous strands (Figure 7CII) as well as associated with the NF^+^ neuronal cell elements (Figure 7CIII). Analysis of IBA1^+^ macrophage interactions with MBP^+^ myelin (Figure 7D) revealed the encroachment of macrophage processes onto node of Ranvier-like structures (Figure 7DI) or spaces (Figure 7DIII) in the ensheathing membrane. IBA1^+^ macrophages with a rounded morphology were also observed interfacing directly with a myelinated axon (Figure 7DII). Three-dimensional reconstructions of confocal image stacks from dual labeling with MBP and IBA1 provided further evidence supporting macrophage interactions with the myelin around the spiral ganglion neuron soma (Figure 7E). These macrophage-myelin interactions are also observed in the osseous spiral lamina, where macrophage processes encircle MBP^+^ myelin elements (shown in Figure 7F), as seen in the apical turn of a 42-year-old donor (H109).

**Figure 8:**
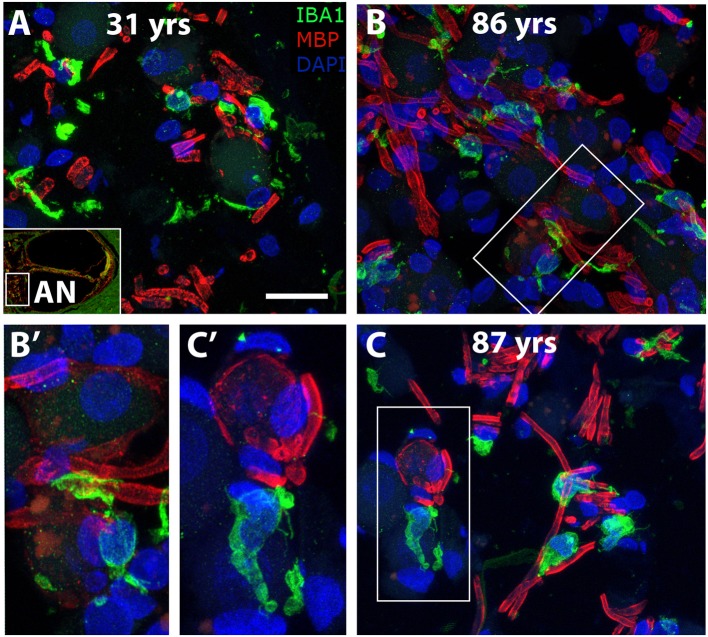
Morphological alterations of macrophages suggest age-related changes in macrophage activity in the human auditory nerve. Dual immunofluorescent staining using antibodies against IBA1 (green) and MBP (red) revealed macrophage associations with the myelin component of axons in a young (**A**, H98) and two older (**B**, H55; **C**, H33) human cochleas. IBA1^+^ immune cell processes appear to encircle the myelin of axonal projections (see enlarged images). Scale bar = 20 μm; 10 μm for enlarged images.

To determine if age-associated alterations in morphology or cellular interactions occur in auditory nerve macrophages, dual labeling immunohistochemistry was applied using antibodies against IBA1 and MBP. Macrophages in the cochlea of a 31-year-old donor (Figure 8A) display an elongated shape, while those in the cochleas from 86- and 87-year-old donors (Figures 8B,C) have a more rounded or ovoid shape. Furthermore, macrophage processes in both older subjects demonstrated ensheathing behavior evidenced by IBA1^+^ processes encompassing myelinated axonal fibers (Figure 8B′). Quantification of IBA1^+^ cells in the middle turn of younger (*n* = 5) vs. older (*n* = 7) cochleas revealed a statistically significant increase in macrophage numbers in the auditory nerve with age (14.1 ± 4.0 vs. = 19.0 ± 3.3 cells; *p* = 0.021). Results of the quantification for each donor sample are presented in Table 3.

## Discussion

Here for the first time we report observations of morphological diversity and age-related changes of macrophages in different regions of the human cochlea. Previous studies have characterized the expression of a number of key cellular markers—MHCII, CD11b, CD68, CD163, and IBA1—in macrophages of the human cochlea (18–20). Additionally, these reports described the morphological heterogeneity of macrophages within the human cochlea. However, none of these studies were able to conduct a quantitative analysis of age-related changes in either morphology or numbers of cochlear macrophages. The specimens used in the present study included 12 human donors from both sexes, with an age gradation of 20–>89-years old allowed analysis of age-related changes in macrophage morphology and numbers in the different cochlear compartments, together with observations of interactions between macrophages and other cell types.

Confocal analysis of frozen sections and whole mount samples from younger ears stained for the macrophage specific marker IBA1 provided evidence to support the morphological diversity of resident immune cells in the lateral wall, as well as in the auditory nerve. In the stria vascularis, macrophages were found to possess diverse cellular phenotypes, reflective of their close interaction with the microvascular network in both mice and humans (34, 35). Here we provide evidence from 12 human temporal bone samples showing that macrophages located in the lateral wall are morphologically distinct from those observed in the auditory nerve. Immune cells in the osseous spiral lamina and Rosenthal's canal were typically “worm-like” in appearance whereas the cyto-architecture of macrophages in the stria vascularis and spiral ligament was more variable and differed from that in the auditory nerve.

It has been well-established that the function of immune cells, especially the macrophage, has a significant impact on its morphology. Macrophages in “surveillance-mode” are typified by a highly branched morphology, while those en route to a location of injury or performing phagocytosis possess an amoeboid shape and the extension of no or only a few cellular processes (33, 36). In the lateral wall, the more prevalent highly ramified cellular morphology of macrophages may be a reflection of their need to receive external stimuli to guide activity, whereas, the “worm-like” structure of the macrophages found in the auditory nerve, is more conducive to motility within this particular cochlear compartment, as suggested by their direct contact with the plasmolemma of glial cells ensheathing axons and neurons which agrees with the direct interaction of macrophages with ganglion neuron cell bodies in mice and humans (29, 37). These differences in morphology likely relate to differences in functional activity in the separate regions of the cochlea.

The diversity in macrophage morphology identified in the different sub-regions of the lateral wall in a single ear may be directly related to the local tissue microenvironment. The suprastrial region (above Reissner's membrane) and the substrial region (below the basilar membrane) of the spiral ligament are largely occupied by type V and IV fibrocytes, respectively, that are bathed in perilymph (38). In these tissue spaces, resident immune cells were predominantly round or elongated in appearance with a few long processes extending from the cell body or, in some instances, more planar with the cytoplasm dispersed uniformly throughout the cell body. It is possible that variations in the architecture of the extracellular matrix or degree of spiral ligament involution could contribute to irregularities in macrophage cell shape (39). Interestingly, the expression pattern of ion transport enzymes by fibrocytes in the spiral ligament has been observed to change relative to the degree of strial atrophy in the aged gerbil cochlea (40).

The cells in the intrastrial space are exposed to an endolymph-like solution, where potassium is sequestered through an electrochemical gradient generated by the parenchymal cells of the stria vascularis and fibrocytes of the spiral ligament. Macrophages found in the intrastrial space are typically ramified in appearance with long cellular processes extending from a central body. In contrast macrophages in the spiral ligament, strial macrophages are typically observed interacting with the microvasculature. While macrophages found in the spiral ligament most likely execute canonical tissue macrophage functions, such as phagocytic uptake and degradation of dead cells and debris, the exact role of intrastrial macrophages remains unknown. The current literature provides conflicting reports regarding macrophage contributions to regulation of blood-labyrinth barrier permeability (34, 41). However, depletion of macrophages during cochlear development in the postnatal mouse results in severe abnormalities of the stria vascularis, implying a role for macrophages in the structural maturation of this specialized epithelium (29). Therefore, further study of changes in macrophage morphology and intercellular dynamics under steady state conditions are needed to understand how this process is regulated.

In this study, results from observations of cochleas from older donors indicated that alterations in macrophage morphology may differ in locations along the tonotopic axis. Interestingly, profiles characteristic of activation were typically observed in the basal turns of the aged cochleas. Quantitative analysis revealed a trending increase in the number of macrophages in the middle and basal turns of the spiral ligament. This increase was accompanied by an increase in the number of macrophages lacking or having very few cellular processes. This finding agrees with observations in mice, showing that a larger proportion of resident immune cells underlying the basilar membrane along the cochlear spiral become more amoeboid in shape with age, indicative of a more active vs. an inactive state (33, 42). To date, *in vitro* evidence of inflammatory cytokine secretion by spiral ligament fibrocytes (43) suggests a possible mechanism for the production of chemotactic factors with age that may induce recruitment of macrophages. Additionally, observations of intravascular monocyte-like cells in the human cochlear lateral wall implicates blood vessels as a likely site of immune cell extravasation (18). In light of these findings, future studies should aim to identify the molecular mediators of macrophage recruitment and infiltration in the aging cochlear lateral wall.

Here we present evidence of macrophage interactions with the glial cell-associated myelinated axonal projections of the type I spiral ganglion neurons and cell bodies in the human cochlea. The increasing frequency of these interactions with age suggests that macrophage activation and abnormal macrophage-glia interactions may be a contributing factor to age-related auditory nerve degeneration. Interestingly, work by Wu et al. (44) assessing cochlear neuropathy in a cohort of 20 human cochleas from “normal-hearing” individuals (0–89 years old), identified the loss of peripherally projecting axonal fibers, which are myelinated by Schwann cells, as a primary pathological process associated with hair cell and spiral ganglion neuron loss. We have shown previously in mice that cochlear macrophages in auditory nerves participate in modulation of glial cell numbers during postnatal development (29). In the present work, we provide evidence of direct interactions of macrophage with the myelinating glia of the auditory nerve in both Rosenthal's canal and the osseous spiral lamina. Quantitative analysis of an age-graded series of human cochleas as performed in this study indicated a statistically significant increase in macrophage numbers in the auditory nerve of the older group, suggesting macrophage activation occurs in the aged auditory nerve. Recent immunohistochemical studies in mice have revealed the complex vascular network associated with the peripheral auditory nervous system, which may be the source of monocyte extravasation to the neural cochlear sub-compartment (45). Further work identifying the molecular components mediating macrophage-glia interactions and structural elements involved in immune cell infiltration are needed to better understand the role of resident and non-resident macrophages in the neural compartment.

“Inflammaging” describes the recently accepted phenomena by which human subjects, typically at the age of 65 and above, present with immune system dysregulation characterized by elevated levels of pro-inflammatory cytokines systemically and an impaired immune response (46–49). A limitation to the current study is the lack of evaluation for infiltration of other inflammatory cell types as lymphocyte infiltration is typically observed in neurodegenerative diseases (42, 50). The presence of CD4^+^ and CD8^+^ T-cells in the peripheral regions of Rosenthal's canal and the spiral ligament of freshly frozen temporal bones has recently been reported (20). Macrophages expressing antigen presentation proteins, such as MHCII, and their interaction with the aforementioned T lymphocytes suggests that adaptive immunity is amongst the cellular immune response processes initiated within the cochlea (20, 51). Nonetheless, the morphological changes and increased numbers of cochlear macrophages observed with age in the lateral wall and auditory nerve is in agreement with functional activation and structural changes of microglia in the central nervous system (52). Future work should address the age-related degenerative pathology occurring in the auditory nerve and the lateral wall focusing on factors which may initiate or exacerbate the potential cochlear inflammaging phenomenon.

## Data Availability

All datasets generated for this study are included in the manuscript and/or the supplementary files.

## Author Contributions

KN, TL, BS, and HL contributed to the conception and design of the study. KN and TL performed the research. KN, HL, TL, BS, and LM analyzed the data. KN wrote the manuscript. All authors discussed the results and contributed to the final manuscript.

### Conflict of Interest Statement

The authors declare that the research was conducted in the absence of any commercial or financial relationships that could be construed as a potential conflict of interest.

## References

[1] Lin FR Niparko JK Ferrucci L. Hearing loss prevalence in the United States. Arch Intern Med. (2011) 171:1851–2. 10.1001/archinternmed.2011.506 22083573 PMC3564588

[2] Dubno JR. Speech recognition across the lifespan: Longitudinal changes from middle age to older adults. Am J Audiol. (2015) 24:84–7. 10.1044/2015_AJA-14-0052 25767998 PMC4610266

[3] Patel R McKinnon BJ. Hearing loss in the elderly. Clin Geriatr Med. (2018) 34:163–74. 10.1016/j.cger.2018.01.001 29661329

[4] Bas E Goncalves S Adams M Dinh CT Bas JM Van De Water TR. Spiral ganglion cells and macrophages initiate neuro-inflammation and scarring following cochlear implantation. Front Cell Neurosci. (2015) 9:303. 10.3389/fncel.2015.00303 26321909 PMC4532929

[5] Latta CH Brothers HM Wilcock DM. Neuroinflammation in Alzheimer's disease; A source of heterogeneity and target for personalized therapy. Neuroscience. (2015) 302:103–11. 10.1016/j.neuroscience.2014.09.061 25286385 PMC4602369

[6] Park I Kassiteridi C Monaco C. Functional diversity of macrophages in vascular biology and disease. Vascul Pharmacol. (2017) 99:13–22. 10.1016/j.vph.2017.10.005 29074468

[7] Friedland DR Cederberg C Tarima S. Audiometric pattern as a predictor of cardiovascular status: development of a model for assessment of risk. Laryngoscope. (2009) 119:473–86. 10.1002/lary.20130 19235737

[8] Ball RY Stowers EC Burton JH Cary NR Skepper JN Mitchinson MJ. Evidence that the death of macrophage foam cells contributes to the lipid core of atheroma. Atherosclerosis. (1995) 114:45–54. 10.1016/0021-9150(94)05463-S 7605375

[9] Loughrey DG Kelly ME Kelley GA Brennan S Lawlor BA. Association of age-related hearing loss with cognitive function, cognitive impairment, and dementia: a systematic review and meta-analysis. JAMA Otolaryngol Head Neck Surg. (2018) 144:115–26. 10.1001/jamaoto.2017.2513 29222544 PMC5824986

[10] Frackowiak J Wisniewski HM Wegiel J Merz GS Iqbal K Wang KC. Ultrastructure of the microglia that phagocytose amyloid and the microglia that produce beta-amyloid fibrils. Acta Neuropathol. (1992) 84:225–33. 10.1007/BF00227813 1414275

[11] Daria A Colombo A Llovera G Hampel H Willem M Liesz A. Young microglia restore amyloid plaque clearance of aged microglia. EMBO J. (2017) 36:583–603. 10.15252/embj.201694591 28007893 PMC5331757

[12] Bredberg G Lindeman HH Ades HW West R Engström H. Scanning electron microscopy of the organ of Corti. Science. (1970) 170:861–3. 10.1126/science.170.3960.861 5482578

[13] Johnsson LG Hawkins JE Jr. Sensory and neural degeneration with aging, as seen in microdissections of the human inner ear. Ann Otol Rhinol Laryngol. (1972) 81:179–93. 10.1177/000348947208100203 4554886

[14] Kusunoki T Cureoglu S Schachern PA Baba K Kariya S Paparella MM. Age-related histopathologic changes in the human cochlea: a temporal bone study. Otolaryngol. Head Neck Surg. (2004) 131:897–903. 10.1016/j.otohns.2004.05.022 15577787

[15] Kidd AR III Bao J Recent advances in the study of age-related hearing loss: a mini-review. Gerontology. (2012) 58:490–6. 10.1159/000338588 22710288 PMC3766364

[16] Makary CA Shin J Kujawa SG Liberman MC Merchant SN. Age-related primary cochlear neuronal degeneration in human temporal bones. J Assoc Res Otolaryngol. (2011) 12:711–7. 10.1007/s10162-011-0283-2 21748533 PMC3214241

[17] Hirose K Rutherford MA Warchol ME. Two cell populations participate in clearance of damaged hair cells from the sensory epithelia of the inner ear. Hear Res. (2017) 352:70–81. 10.1016/j.heares.2017.04.006 28526177 PMC5544544

[18] Liu W Molnar M Garnham C Benav H Rask-Andersen H. Macrophages in the human cochlea: saviors or predators-a study using super-resolution immunohistochemistry. Front Immunol. (2018) 9:223. 10.3389/fimmu.2018.00223 29487598 PMC5816790

[19] O'Malley JT Nadol JB Jr McKenna MJ. Anti CD163+, Iba1+, and CD68+ cells in the adult human inner ear: normal distribution of an unappreciated class of macrophages/microglia and implications for inflammatory otopathology in humans. Otol Neurotol. (2016) 37:99–108. 10.1097/MAO.0000000000000879 26485593 PMC4675683

[20] Liu W Rask-Andersen H. Super-resolution immunohistochemistry study on CD4 and CD8 cells and the relation to macrophages in human cochlea. J Otol. (2019) 14:1–5. 10.1016/j.joto.2018.11.010 30936894 PMC6424713

[21] Shigemoto-Mogami Y Hoshikawa K Sato K. Activated microglia disrupt the blood-brain barrier and induce chemokines and cytokines in a rat *in vitro* model. Front Cell Neurosci. (2018) 12:494. 10.3389/fncel.2018.00494 30618641 PMC6300509

[22] Liddelow SA Guttenplan KA Clarke LE Bennett FC Bohlen CJ Schirmer L. Neurotoxic reactive astrocytes are induced by activated microglia. Nature. (2017) 541:481–7. 10.1038/nature21029 28099414 PMC5404890

[23] Schuknecht H. Temporal bone removal at autopsy. Preparation and uses. Arch Otolaryngol. (1968) 87:129–37. 10.1001/archotol.1968.00760060131007 4865202

[24] Xing Y Samuvel DJ Stevens SM Dubno JR Schulte BA Lang H. Age-related changes of myelin basic protein in mouse and human auditory nerve. PLoS ONE. (2012) 7:e34500. 10.1371/journal.pone.0034500 22496821 PMC3320625

[25] Hao X Xing Y Moore MW Zhang J Han D Schulte BA. Sox10 expressing cells in the lateral wall of the aged mouse and human cochlea. PLoS ONE. (2014) 9:e97389. 10.1371/journal.pone.0097389 24887110 PMC4041576

[26] Cunningham CD III Weber PC Spicer SS Schulte BA. Canalicular reticulum in vestibular hair cells. Hear Res. (2000) 143:69–83. 10.1016/S0378-5955(00)00022-8 10771185

[27] Weber PC Cunningham CD III Schulte BA. Potassium recycling pathways in the human cochlea. Laryngoscope. (2001) 111:1156–65. 10.1097/00005537-200107000-00006 11568535

[28] Viana LM O'Malley JT Burgess BJ Jones DD Oliveira CA Santos F. Cochlear neuropathy in human presbycusis: confocal analysis of hidden hearing loss in post-mortem tissue. Hear Res. (2015) 327:78–88. 10.1016/j.heares.2015.04.014 26002688 PMC4554812

[29] Brown LN Xing Y Noble KV Barth JL Panganiban CH Smythe NM. Macrophage-mediated glial cell elimination in the postnatal mouse cochlea. Front Mol Neurosci. (2017) 10:407. 10.3389/fnmol.2017.00407 29375297 PMC5770652

[30] Liu T Li G Noble KV Li Y Barth JL Schulte BA. Age-dependent alterations of Kir4.1 expression in neural crest-derived cells of the mouse and human cochlea. Neurobiol Aging. (2019) 80:210–22. 10.1016/j.neurobiolaging.2019.04.009 31220650 PMC6679794

[31] Van den Heuvel MM Tensen CP van As JH Van den Berg TK Fluitsma DM Dijkstra CD. Regulation of CD 163 on human macrophages: cross-linking of CD163 induces signaling and activation. J Leukoc Biol. (1999) 66:858–66. 10.1002/jlb.66.5.858 10577520

[32] Van Gorp H Delputte PL Nauwynck HJ. Scavenger receptor CD163, a Jack-of-all-trades and potential target for cell-directed therapy. Mol Immunol. (2010) 47:1650–60. 10.1016/j.molimm.2010.02.008 20299103

[33] Stence N Waite M Dailey ME. Dynamics of microglial activation: a confocal time-lapse analysis in hippocampal slices. Glia. (2001) 33:256–66. 10.1002/1098-1136(200103)33:3<256::AID-GLIA1024>3.3.CO;2-A 11241743

[34] Zhang W Dai M Fridberger A Hassan A Degagne J Neng L. Perivascular-resident macrophage-like melanocytes in the inner ear are essential for the integrity of the intrastrial fluid-blood barrier. Proc Natl Acad Sci USA. (2012) 109:10388–93. 10.1073/pnas.1205210109 22689949 PMC3387119

[35] Shi X. Resident macrophages in the cochlear blood-labyrinth barrier and their renewal via migration of bone-marrow-derived cells. Cell Tissue Res. (2010) 342:21–30. 10.1007/s00441-010-1040-2 20838812

[36] Leone C Le Pavec G Même W Porcheray F Samah B Dormont D. Characterization of human monocyte-derived microglia-like cells. Glia. (2006) 54:183–92. 10.1002/glia.20372 16807899

[37] Szepesi Z Manouchehrian O Bachiller S Deierborg T. Bidirectional microglia-neuron communication in health and disease. Front Cell Neurosci. (2018) 12:323. 10.3389/fncel.2018.00323 30319362 PMC6170615

[38] Spicer SS Schulte BA. Differentiation of inner ear fibrocytes according to their ion transport related activity. Hear Res. (1991) 56:53–64. 10.1016/0378-5955(91)90153-Z 1663106

[39] Spicer SS Schulte BA. Spiral ligament pathology in quiet-aged gerbils. Hear Res. (2002) 172:172–85. 10.1016/S0378-5955(02)00581-6 12361880

[40] Spicer SS Gratton MA Schulte BA. Expression patterns of ion transport enzymes in spiral ligament fibrocytes change in relation to strial atrophy in the aged gerbil cochlea. Hear Res. (1997) 111:93–102. 10.1016/S0378-5955(97)00097-X 9307315

[41] Hirose K Li SZ. The role of monocytes and macrophages in the dynamic permeability of the blood-perilymph barrier. Hear Res. (2019) 374:49–57. 10.1016/j.heares.2019.01.006 30710792 PMC6459018

[42] Raivich G Bohatschek M Kloss CU Werner A Jones LL Kreutzberg GW. Neuroglial activation repertoire in the injured brain: graded response, molecular mechanisms and cues to physiological function. Brain Res Rev. (1999) 30:77–105. 10.1016/S0165-0173(99)00007-7 10407127

[43] Ichimiya I Yoshida K Hirano T Suzuki M Mogi G. Significance of spiral ligament fibrocytes with cochlear inflammation. Int J Pediatr Otorhinolaryngol. (2000) 56:45–51. 10.1016/S0165-5876(00)00408-0 11074115

[44] Wu PZ Liberman LD Bennett K de Gruttola V O'Malley JT Liberman MC. (2019). Primary neural degeneration in the human cochlea: evidence for hidden hearing loss in the aging ear. Neuroscience. 407:8–20. 10.1016/j.neuroscience.2018.07.053 30099118 PMC6369025

[45] Jiang H Wang X Zhang J Kachelmeier A Lopez IA Shi X. Microvascular networks in the area of the auditory peripheral nervous system. Hear Res. (2019) 371:105–16. 10.1016/j.heares.2018.11.012 30530270

[46] Bruunsgaard H Andersen-Ranberg K Jeune B Pedersen AN Skinhøj P Pedersen BK. A high plasma concentration of TNF-alpha is associated with dementia in centenarians. J Gerontol A Biol Sci Med Sci. (1999) 54:M357–64. 10.1093/gerona/54.7.M357 10462168

[47] Fagiolo U Cossarizza A Scala E Fanales-Belasio E Ortolani C Cozzi E. Increased cytokine production in mononuclear cells of healthy elderly people. Eur J Immunol. (1993) 23:2375–8. 10.1002/eji.1830230950 8370415

[48] Mari D Mannucci PM Coppola R Bottasso B Bauer KA Rosenberg RD. Hypercoagulability in centenarians: the paradox of successful aging. Blood. (1995) 85:3144–9. 7756646

[49] Ferrucci L Harris TB Guralnik JM Tracy RP Corti MC Cohen HJ. Serum IL-6 level and the development of disability in older persons. J Am Geriatr Soc. (1999) 47:639–46. 10.1111/j.1532-5415.1999.tb01583.x 10366160

[50] Kawamata T Akiyama H Yamada T McGeer PL. Immunologic reactions in amyotrophic lateral sclerosis brain and spinal cord tissue. Am J Pathol. (1992) 140:691–707. 1347673 PMC1886170

[51] Kämpfe Nordström C Danckwardt-Lillieström N Laurell G Liu W Rask-Andersen H The human endolymphatic sac and inner ear immunity: macrophage interaction and molecular expression. Front Immunol. (2018) 9:3181 10.3389/fimmu.2018.03181 30774637 PMC6367985

[52] Davis EJ Foster TD Thomas WE. Cellular forms and functions of brain microglia. Brain Res Bull. (1994) 34:73–8. 10.1016/0361-9230(94)90189-9 8193937

